# Label-free mass spectrometric analysis reveals complex changes in the brain proteome from the *mdx*-*4cv* mouse model of Duchenne muscular dystrophy

**DOI:** 10.1186/s12014-015-9099-0

**Published:** 2015-11-23

**Authors:** Sandra Murphy, Margit Zweyer, Michael Henry, Paula Meleady, Rustam R. Mundegar, Dieter Swandulla, Kay Ohlendieck

**Affiliations:** Department of Biology, Maynooth University, National University of Ireland, Maynooth, Co. Kildare Ireland; Department of Physiology II, University of Bonn, 53115 Bonn, Germany; National Institute for Cellular Biotechnology, Dublin City University, Dublin 9, Ireland

**Keywords:** Annexin, Ca^2+^-ATPase PMCA2, Dystrophinopathy, Glial fibrillary acidic protein, Glyosis, Intermediate filament, Mental retardation, Vimentin, von Willebrand factor

## Abstract

**Background:**

X-linked muscular dystrophy is a primary disease of the neuromuscular system. Primary abnormalities in the *Dmd* gene result in the absence of the full-length isoform of the membrane cytoskeletal protein dystrophin. Besides progressive skeletal muscle wasting and cardio-respiratory complications, developmental cognitive deficits and behavioural abnormalities are clinical features of Duchenne muscular dystrophy. In order to better understand the mechanisms that underlie impaired brain functions in Duchenne patients, we have carried out a proteomic analysis of total brain extracts from the *mdx*-*4cv* mouse model of dystrophinopathy.

**Results:**

The comparative proteomic profiling of the *mdx*-*4cv* brain revealed a significant increase in 39 proteins and a decrease in 7 proteins. Interesting brain tissue-associated proteins with an increased concentration in the *mdx*-*4cv* animal model were represented by the glial fibrillary acidic protein GFAP, the neuronal Ca^2+^-binding protein calretinin, annexin AnxA5, vimentin, the neuron-specific enzyme ubiquitin carboxyl-terminal hydrolase isozyme L1, the dendritic spine protein drebrin, the cytomatrix protein bassoon of the nerve terminal active zone, and the synapse-associated protein SAP97. Decreased proteins were identified as the nervous system-specific proteins syntaxin-1B and syntaxin-binding protein 1, as well as the plasma membrane Ca^2+^-transporting ATPase PMCA2 that is mostly found in the brain cortex. The differential expression patterns of GFAP, vimentin, PMCA2 and AnxA5 were confirmed by immunoblotting. Increased GFAP levels were also verified by immunofluorescence microscopy.

**Conclusions:**

The large number of mass spectrometrically identified proteins with an altered abundance suggests complex changes in the *mdx*-*4cv* brain proteome. Increased levels of the glial fibrillary acidic protein, an intermediate filament component that is uniquely associated with astrocytes in the central nervous system, imply neurodegeneration-associated astrogliosis. The up-regulation of annexin and vimentin probably represent compensatory mechanisms involved in membrane repair and cytoskeletal stabilization in the absence of brain dystrophin. Differential alterations in the Ca^2+^-binding protein calretinin and the Ca^2+^-pumping protein PMCA2 suggest altered Ca^2+^-handling mechanisms in the Dp427-deficient brain. In addition, the proteomic findings demonstrated metabolic adaptations and functional changes in the central nervous system from the dystrophic phenotype. Candidate proteins can now be evaluated for their suitability as proteomic biomarkers and their potential in predictive, diagnostic, prognostic and/or therapy-monitoring approaches to treat brain abnormalities in dystrophinopathies.

## Background

Proteome-wide studies play a central role in the systematic assessment of diseases of childhood. Biomedical surveys based on mass spectrometry-based proteomics can provide a comprehensive overview of molecular changes that underlie paediatric disorders and identify novel proteomic biomarker candidates for improving predictive, diagnostic, prognostic and therapy-monitoring procedures [1–3]. Duchenne muscular dystrophy is a paediatric disease of the skeletal musculature that is characterized by a variety of abnormalities in muscle tissues, including changes in myofibre size, central nucleation, fibre branching, hyper-contractility, necrosis, inflammation, fatty deposition and myofibrosis [4–6]. Mutations or genetic rearrangements in the X-chromosomal *Dmd* gene encoding the membrane cytoskeletal protein dystrophin are the underlying cause for highly progressive skeletal muscle wasting [7]. The reduction in dystrophin-associated glycoproteins is a hallmark of fibre degeneration and closely linked to the loss of sarcolemmal integrity in muscular dystrophy [8–10]. Associated complications in Duchenne patients are cardio-respiratory impairments, orthopaedic problems causing muscle contractures and scoliosis, endocrinological issues related to growth and weight gain, as well as gastrointestinal, renal, urinary and ophthalmological complications [11–14] that are taken into account in the current treatment and management of dystrophinopathies [15–17].

The elucidation of the molecular and cellular mechanisms of the multi-systemic manifestation of Duchenne muscular dystrophy in non-muscle tissues is complicated by the existence of several promoters that drive the tissue-specific expression of dystrophin isoforms ranging in molecular mass from 71 to 427 kDa [18]. Brain dystrophins and their associated glycoproteins are mainly involved in neuronal excitability, signal integration, synaptic modulation and neuronal plasticity [19–22]. In the nervous system, major dystrophin isoforms include Dp71, Dp140 and Dp427 [23]. Full-length brain dystrophins are present in neurons of the cerebral cortex and the hippocampus, as well as in cerebellar Purkinje cells [24–27] and exhibit similar biochemical properties as the muscle Dp427 isoform [28, 29]. The shorter dystrophin isoform Dp140 is most highly expressed during brain development [30] and the most abundant brain dystrophin, Dp71, is present in both neurons and glia cells in the *dentate gyrus* and the olfactory bulb [31–33].

The presence of specific dystrophins in the central nervous system is of considerable interest, since cognitive impairments and emotional disturbances are established clinical features of dystrophinopathies [34–36]. Mental retardation and behavioural impairments seem to be secondary to physical handicap [37–40] and do not correlate with the progressive nature of the neuromuscular pathology in X-linked muscular dystrophy [41–44]. Cognitive impairments seem to affect memory, attention, language and emotion to a differing degree in individuals suffering from dystrophinopathies [45–47]. In analogy to Duchenne patients, the *mdx* animal model of dystrophinopathy shows significant alterations in associative learning patterns and deficits in long-term consolidation memory [48–50], as well as metabolic and cellular abnormalities in distinct brain regions [51–53].

In order to evaluate the degree of proteome-wide changes in the central nervous system of the dystrophin-deficient mouse, we have carried out a comparative label-free mass spectrometric analysis of the *mdx*-*4cv* brain versus wild type brain. Systematic proteomic studies have previously established a number of changes in proteins involved in energy metabolism, cellular signalling, the extracellular matrix, cytoskeletal networks and the cellular stress response in dystrophic skeletal and cardiac muscles [54]. Here, we have applied this technology-driven approach to extend these studies to the pathophysiological mechanisms that facilitate impaired brain functions in X-linked muscular dystrophy. Highly relevant proteins affected in brain tissue from dystrophic mice were identified as the glial fibrillary acidic protein GFAP, calretinin, annexin AnxA5, vimentin, syntaxin, drebrin, bassoon and the plasma membrane Ca^2+^-ATPase PMCA2.

## Results and discussion

Intellectual impairments and emotional disturbances are clearly present in Duchenne patients [45], although these clinical features are non-progressive and do not affect all dystrophic children [37]. Besides the unknown mechanisms that underlie this differential occurrence of neurological issues within the Duchenne patient cohort, other crucial unanswered questions remain in relation to the molecular and cellular pathogenesis of brain abnormalities. Importantly, it is not currently clear whether a pathophysiological hierarchy exists between developmental issues on the one hand and cycles of neurodegeneration and astroglyosis on the other hand in the central nervous system of patients afflicted with X-linked muscular dystrophy [46]. It was therefore of interest to use an unbiased and technology-driven approach to attempt a systematic evaluation of proteome-wide changes in affected brain tissue. In general, comparative proteomics has the potential to identify complex changes within cellular systems on a more global basis as compared to individual and hypothesis-driven biochemical, cell biological or physiological investigations. The proteomic profiling of the *mdx*-*4cv* brain presented here has successfully detected significant changes in a number of representative serum and brain proteins.

### Label-free LC–MS/MS analysis of mouse brain extracts

**Fig. 1:**
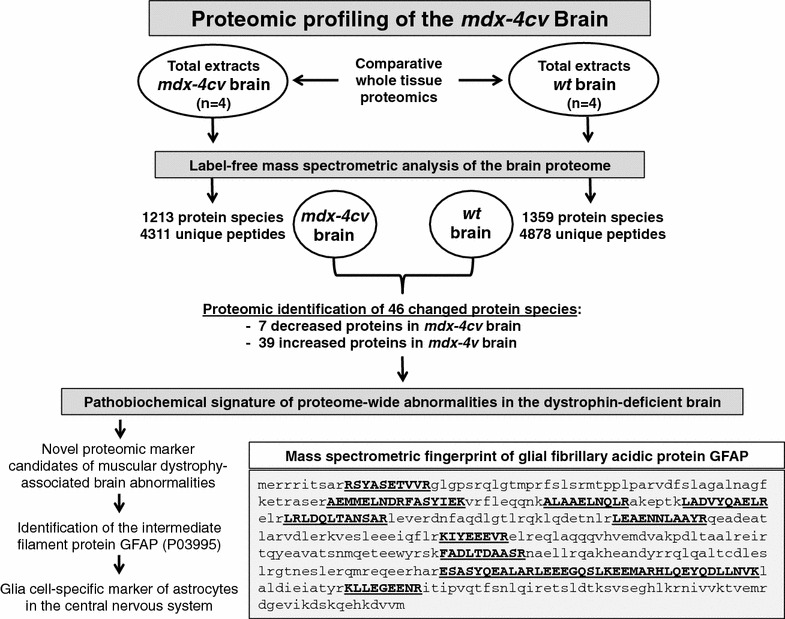
Overview of the proteomic strategy to determine global changes in the protein constellation of the *mdx*-*4cv* brain. The *flow chart* outlines the comparative analysis of wild type versus *mdx*-*4cv* brain extracts and shows the mass spectrometric fingerprint of the glial fibrillary acidic protein GFAP as a major finding of this proteomic study

An overview of the comparative proteomic analysis of the wild type brain versus the *mdx*-*4cv* brain employing label-free mass spectrometry is given in Fig. 1. The proteomic assessment of the mouse brain identified 4878 unique peptides belonging to 1359 individual protein species in the wild type and 4311 unique peptides belonging to 1213 individual protein species in the *mdx*-*4cv* phenotype. The fact that this technique was successfully applied to study the mouse brain proteome is exemplified by the mass spectrometric identification of 2 well-established brain proteins, BASP-1 and SNP-25. The growth-associated brain acid soluble protein BASP-1 (Q91XV3; also known as neuronal axonal membrane protein NAP-22) is a membrane-attached signalling protein of growth cones that form the tips of elongating axons [55]. Its sequence was covered in the wild type brain by 16 unique peptides with 87 % coverage and in the *mdx*-*4cv* brain by 17 unique peptides with 87 % coverage. The synaptosome-associated protein SNP-25 (P60879) is associated with proteins involved in vesicle docking and membrane fusion and plays a critical role in synaptic function and the molecular regulation of neurotransmitter release [56]. SNP-25 was covered in the wild type brain by 13 unique peptides with 47 % coverage and in the *mdx*-*4cv* brain by 11 unique peptides with 46 % coverage. In contrast to gel-based studies, the usage of liquid chromatography in combination with highly sensitive label-free mass spectrometry identified both extremely large proteins and highly hydrophobic integral membrane proteins. These types of brain proteins are usually underrepresented in two-dimensional gel electrophoresis. For example, the sequence of the 500-kDa intermediate filament-binding protein plectin (Q9QXS1) [57] was covered in the wild type brain by 18 unique peptides with approximately 5 % coverage and in the *mdx*-*4cv* brain by 17 unique peptides with approximately 4 % coverage. Remarkably, the five main subunits of the plasmalemmal Na^+^/K^+^-ATPase [58] were all identified in brain extracts. The alpha-1 (Q8VDN2), alpha-2 (Q6PIE5), alpha-3 (Q6PIC6), beta-1 (P14094) and beta-2 (P14231) subunits were covered in the wild type brain by 13, 17, 20, 9 and 4 unique peptides with 20, 21, 27, 19 and 10 % coverage, respectively. These findings demonstrate the outstanding performance of the LTQ Orbitrap XL mass spectrometer used in this comparative whole tissue proteomic study.

### Label-free LC–MS/MS analysis of changed proteins in total *mdx*-*4cv* brain extracts

The proteomic comparison of total brain extracts from wild type versus *mdx*-*4cv* mice using label-free LC–MS/MS analysis identified 46 affected protein species in the dystrophic phenotype (Fig. 1). Table 1 lists the accession number, protein name, peptide count, confidence score, Anova value and fold change of individual proteins identified by mass spectrometry. An increased abundance was established for 39 proteins and 7 proteins showed a decreased concentration. Of considerable interest was the proteomic identification of elevated serum proteins, especially the confirmation of previously established biomarkers of muscular dystrophy. Increased proteins that are mostly associated with plasma and the extracellular space were identified as hemopexin (beta-1B-glycoprotein), alpha-2-HS-glycoprotein (fetuin-A), alpha-1-antitrypsin 1-1 and 1-2, hemoglobin alpha and beta-1, apolipoprotein A-I, albumin and serotransferrin. The increase of this class of soluble protein is most likely due to disease-related changes in the concentration levels of distinct plasma proteins, but could also be partially associated with altered capillarisation, local cellular damage, disturbed homeostasis, impaired regulatory mechanisms, proteolytic processes and/or inflammatory responses.

**Table 1:** Changed proteins in *mdx*-*4cv* brain tissue as revealed by label-free LC–MS/MS analysis

Accession number	Protein name	Peptide count	Confidence score	Anova (p)	Fold change
Q91X72	Hemopexin	3	131.20	0.00116	5.64
P29699	Alpha-2-HS-glycoprotein	2	125.19	0.00171	3.47
P22599	Alpha-1-antitrypsin 1-2	2	114.76	0.01445	3.29
P02088	Hemoglobin subunit beta-1	3	260.19	0.00104	3.24
P01942	Hemoglobin subunit alpha	5	370.80	0.00021	3.22
Q00623	Apolipoprotein A-I	8	316.96	0.00522	2.88
P07758	Alpha-1-antitrypsin 1-1	4	108.27	0.01739	2.52
P07724	Serum albumin	16	1174.47	0.00049	2.51
Q923D2	Flavin reductase (NADPH)	3	203.95	0.00171	2.51
Q921I1	Serotransferrin	8	374.58	0.01661	2.18
P03995	Glial fibrillary acidic protein	8	491.51	0.03531	2.00
Q08331	Calretinin	2	94.24	0.04022	1.93
P48036	Annexin A5	2	132.70	0.03204	1.56
P20152	Vimentin	2	213.31	0.00440	1.55
Q9R0P9	Ubiquitin carboxyl-terminal hydrolase isozyme L1	3	165.79	0.02308	1.51
P46460	Vesicle-fusing ATPase	2	134.05	0.00110	1.44
Q9D0E1	Heterogeneous nuclear ribonucleoprotein M	3	63.46	0.01490	1.44
O08788	Dynactin subunit 1	2	126.53	0.01025	1.44
P38647	Stress-70 protein, mitochondrial	6	476.79	0.00175	1.42
P48758	Carbonyl reductase [NADPH] 1	2	119.09	0.01333	1.42
P00920	Carbonic anhydrase 2	2	135.96	0.02814	1.42
P14733	Lamin-B1	2	150.58	0.02918	1.41
Q6IRU5	Clathrin light chain B	2	125.00	0.04203	1.40
Q8R3V5	Endophilin-B2	4	201.90	0.02055	1.39
Q8QZT1	Acetyl-CoA acetyltransferase, mitochondrial	4	215.23	0.00680	1.36
P27773	Protein disulfide-isomerase A3	5	295.10	0.01295	1.35
Q91VR2	ATP synthase subunit gamma, mitochondrial	4	251.82	0.00255	1.35
Q6ZWY9	Histone H2B type 1-C/E/G	2	121.60	0.04212	1.34
Q61171	Peroxiredoxin-2	2	141.40	0.01279	1.33
Q9D051	Pyruvate dehydrogenase E1 component subunit beta, mitochondrial	4	262.12	0.00408	1.32
O35737	Heterogeneous nuclear ribonucleoprotein H	2	100.27	0.01665	1.31
Q03265	ATP synthase subunit alpha, mitochondrial	6	480.06	0.00581	1.30
P20029	78 kDa glucose-regulated protein	2	150.07	0.02510	1.29
Q9QXS6	Drebrin	3	142.38	0.01934	1.28
P62259	14-3-3 protein epsilon	2	103.43	0.02864	1.27
O88737	Protein bassoon	3	215.46	0.01328	1.25
Q811D0	Disks large homolog 1	2	110.76	0.00639	1.25
P14873	Microtubule-associated protein 1B	2	126.07	0.00500	1.24
P16546	Spectrin alpha chain, non-erythrocytic 1	3	197.38	0.01491	1.24
P61264	Syntaxin-1B	4	356.83	0.00121	−1.28
Q02053	Ubiquitin-like modifier-activating enzyme 1	2	131.59	0.04436	−1.41
P51150	Ras-related protein Rab-7a	3	106.89	0.01101	−1.51
P07901	Heat shock protein HSP 90-alpha	2	109.14	0.03836	−1.54
O08599	Syntaxin-binding protein 1	4	315.93	0.01966	−1.54
Q9WV92	Band 4.1-like protein 3	3	218.67	0.00892	−1.90
Q9R0K7	Plasma membrane calcium-transporting ATPase 2	2	108.25	0.01784	−2.77

**Fig. 2:**
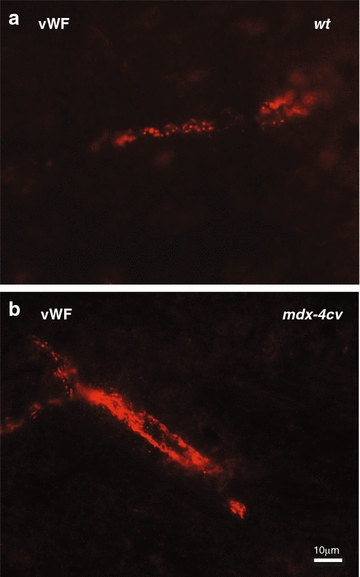
Immunofluorescence microscopical analysis of von Willebrand factor in normal versus *mdx*-*4cv* brain. Shown is the immunofluorescence labelling of brain sections using an antibody to von Willebrand factor (vWF) in wild type **a** versus *mdx*-*4cv* **b** cortex. *Dot-like* staining patterns are seen in both normal and *mdx*-*4cv* brain sections and represent Weibel-Palade bodies [61] that function as intracellular storage organelles for vWF molecules in the vessel endothelium. The *mdx*-*4cv* brain shows also a large number of string-like structures that contain vWF molecules, which is indicative of structural alterations, vascular damage and inflammatory processes at the blood-tissue interface in the *mdx*-*4cv* brain. *Bar* equals 10 μm

Immunofluorescence microscopy of brain sections using an antibody to von Willebrand factor (vWF) indicated changes in the vascularization of the *mdx*-*4cv brain* (Fig. 2). The dot-shaped labelling of Weibel-Palade bodies [59], that act as specific intracellular storage organelles for vWF molecules in the vessel endothelium [60], can be clearly seen as distinct subcellular structures in normal brain sections (Fig. 2a). Under normal conditions, multimeric vWF molecules assemble into tubular structures within Weibel-Palade bodies in the inner lining of blood vessels. However, when vWF molecules are released they unfold into strings and play a critical role in hemostasis, inflammation, regulation of vascular tone and angiogenesis [61]. The *mdx*-*4cv* brain showed, besides punctate Weibel-Palade bodies, also a large number of string-like structures that contain vWF molecules (Fig. 2b). Since this type of transformation is indicative of a response of the endothelium to pathophysiological changes in its micro environment [60], dystrophin deficiency appears to trigger the subcellular translocation of Weibel-Palade bodies and the release of vWF. Therefore structural distension, vascular damage and inflammatory processes appear to exist at the blood-tissue interface in the *mdx*-*4cv* brain. This would agree with the cell biological analysis of tight junction and glial markers in the *mdx* brain, which showed that microvessels are lined by altered endothelial cells with open tight junctions and swollen glial processes. The perivascular glial endfeet zone was shown to contain cellular debris and was enveloped by degenerating microvessels [62].

The approximately fivefold increase of hemopexin, an abundant serum glycoprotein that binds free heme, is a well-established disease indicator in the plasma from Duchenne muscular dystrophy patients and carriers of this neuromuscular disorder [63, 64]. Since serotransferrin is responsible for the efficient binding and transportation of iron throughout the circulatory system [65], its increased concentration could be linked to an altered iron metabolism in the *mdx*-*4cv* brain. The elevated levels of haemoglobin and albumin suggest a potential shift to higher levels of oxygen supply and fatty acid utilization. Interestingly, antitrypsin appears to be increased in brain tissue. This serum protein functions both as a protective anti-protease and a crucial anti-inflammatory factor [66], suggesting the triggering of a protective mechanism within Dp427-lacking tissue in response to neuronal degeneration and brain inflammation. The identification of fetuin-A also agrees with the pathological status of the *mdx*-*4cv* mouse, since this multi-functional protein has been previously identified as a general biomarker for neurodegenerative disease [67].

### Altered brain tissue-associated proteins in the *mdx*-*4cv* mouse model of dystrophinopathy

Interesting brain tissue-associated proteins with an increased concentration in the *mdx*-*4cv* animal model of Duchenne muscular dystrophy were represented by flavin reductase, glial fibrillary acidic protein GFAP, the neuronal Ca^2+^-binding protein calretinin, annexin AnxA5, vimentin, the neuron-specific enzyme ubiquitin carboxyl-terminal hydrolase isozyme L1, vesicle-fusing ATPase, heterogeneous nuclear ribonucleoproteins M and H, dynactin, the 75 and 78 kDa glucose-regulated proteins (mitochondrial chaperones HspA9/GRP-75 and HspA5/Grp78), carbonyl reductase, carbonic anhydrase CA2, lamin-B1, clathrin, endophilin-B2, mitochondrial acetyl-CoA acetyltransferase, protein disulfide-isomerase PDI-A3, mitochondrial ATP synthase (alpha and gamma subunits), histone H2B, peroxiredoxin PRDX2, mitochondrial pyruvate dehydrogenase, the dendritic spine protein drebrin, 14-3-3 protein epsilon, the cytomatrix protein bassoon of the nerve terminals active zone, the synapse-associated protein SAP97 (disks large homolog 1), microtubule-associated protein 1B and spectrin (Table 1). Decreased brain proteins were identified as the nervous system-specific proteins syntaxin-1B and syntaxin-binding protein 1, as well as the plasma membrane Ca^2+^-transporting ATPase PMCA2 that is mostly found in the brain cortex. Other reduced brain-associated proteins included ubiquitin-like modifier-activating enzyme 1, ras-related protein Rab-7a, heat shock protein HSP 90-alpha (HSP90AA1) and 4.1-like protein 3 (Table 1).

### Distribution of protein changes in *mdx*-*4cv* brain tissue

**Fig. 3:**
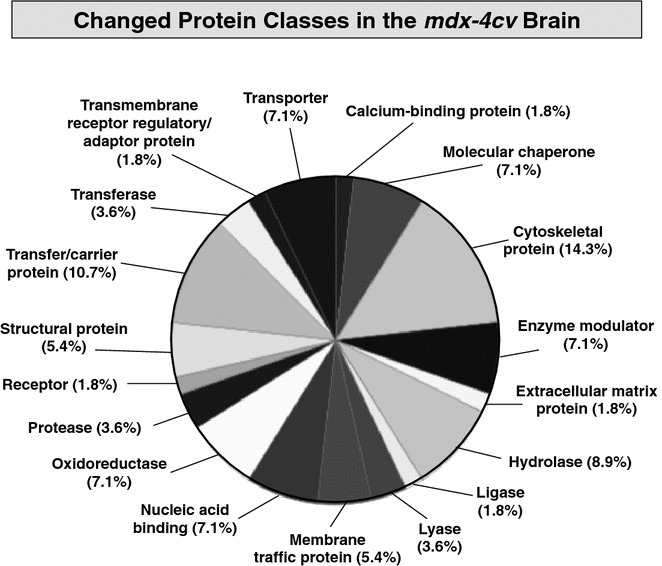
Overview of changed protein classes in total tissue extracts from the *mdx*-*4cv* brain. In order to identify the clustering of protein classes based on the mass spectrometric analysis of crude extracts from wild type versus *mdx*-*4cv* brain specimens (Table 1), the bioinformatics software programme PANTHER [68] was used

The distribution pattern of proteome-wide changes in *mdx*-*4cv* brain tissue in relation to altered protein classes is shown in Fig. 3. A standard bioinformatics software programme was used to illustrate overall protein changes. The application of the PANTHER analysis tool [68] revealed a certain degree of clustering of mass spectrometrically identified proteins with a changed concentration in *mdx*-*4cv* brain tissues. The most drastic alterations were shown to affect cytoskeletal proteins (14.3 %), which would agree with the underlying primary genetic abnormality in the membrane cytoskeletal component dystrophin. Considerable changes were shown to occur in the molecular classes of transfer/carrier proteins (10.7 %), hydrolases (8.9 %), enzyme modulators (7.1 %), molecular chaperones (7.1 %), transporters (7.1 %), oxidoreductases (7.1 %), nucleic acid binding proteins (7.1 %), membrane traffic proteins (5.4 %) and structural proteins (5.4 %). Relatively minor variations were identified for proteases (3.6 %), transferases (3.6 %), lyases, (3.6 %), receptors (1.8 %), transmembrane receptor regulatory/adaptor proteins (1.8 %), Ca^2+^-binding proteins (1.8 %), extracellular matrix proteins (1.8 %) and ligases (1.8 %). Select proteomic findings were further analysed by immunoblotting and immunofluorescence microscopy in order to independently verify the results from the comparative label-free LC–MS/MS study of brain tissue extracts from wild type versus *mdx*-*4cv* mice.

### Elevated levels of glial fibrillary acidic protein agrees with the pathobiochemical concept of neurodegeneration-related astrogliosis in the *mdx*-*4cv* brain

**Fig. 4:**
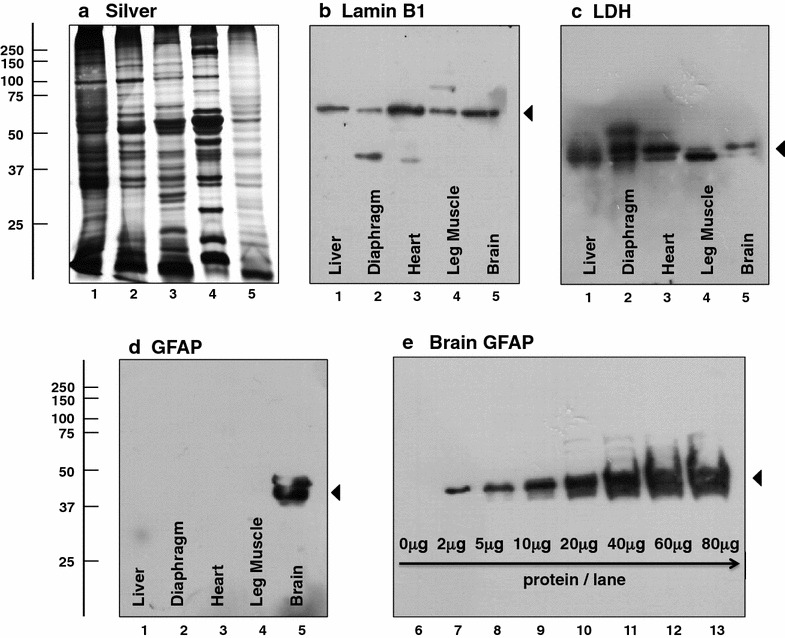
Immunoblot analysis of the tissue-specific expression of the glial fibrillary acidic protein GFAP. Shown is a silver-stained gel (**a**) and corresponding immunoblots labelled with antibodies to the nuclear envelope protein lamin B1 (**b**), the cytosolic enzyme lactate dehydrogenase (**c**; LDH) and the glial fibrillary acidic protein (**d**; GFAP). *Lanes 1*–*5* contain crude extracts from mouse liver, diaphragm, heart, leg muscle and brain, respectively. While lamin and lactate dehydrogenase were detected in all five tissues studied, GFAP could only be detected in brain samples, demonstrating the tissue-specific recognition of this protein by the antibody ab7260 to GFAP. Antibody labelling of increasing amounts of mouse brain protein per gel lane showed that a broader immuno-labelled GFAP banding pattern occurs at higher protein amounts (**e**). *Lanes* *6*–*13* contained 0, 2, 5, 10, 20, 40, 60 and 80 μg protein/lane, respectively

The twofold increase of the glial fibrillary acidic protein (Table 1), which was identified by 48 % sequence coverage (Fig. 1), is an interesting proteomic finding of considerable pathobiochemical importance [69]. This brain protein is often referred to as GFAP [70] and functions as a major intermediate filament component that is specifically associated with astrocytes in the central nervous system [71]. Importantly, an increased concentration of GFAP is an established indicator of glial scar formation in the field of neuropathology [72–74]. The independent verification of expression changes in GFAP in *mdx*-*4cv* brain tissue was carried out by both immunoblot analysis and immunofluorescence microscopy. Figure 4 illustrates the specificity of the antibody to GFAP used in immunoblotting. While the nuclear envelope marker lamin and the cytosolic enzyme lactate dehydrogenase are present in crude extracts from mouse liver, diaphragm, heart, leg muscles and brain tissue (Fig. 4b, c), GFAP was only detected in brain specimens (Fig. 4d). A silver-stained gel of the protein extracts from different tissues is presented in Fig. 4a. The immuno-labelling of GFAP molecules often results in the recognition of more than one band in one-dimensional gel electrophoresis. An immunoblot study of GFAP by Singh et al. [75] has shown a tight banding pattern of this glial marker with 2–3 protein bands with slightly differing electrophoretic mobility. In analogy, the antibody decoration of increasing amounts of mouse brain protein per gel lane, as shown in Fig. 4e, demonstrated that a broader immuno-labelled GFAP banding pattern occurs at higher protein amounts. Based on these findings, an intermediate amount of brain protein was used per lane for comparative studies.

**Fig. 5:**
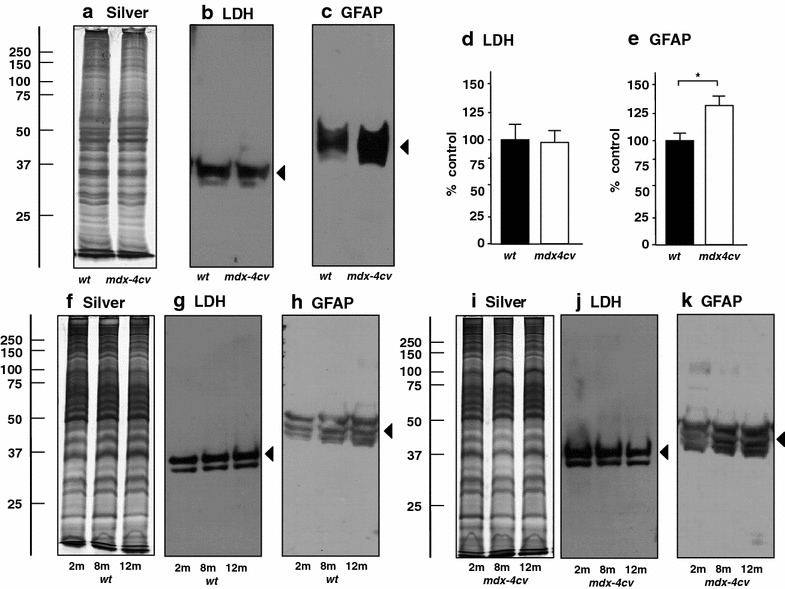
Comparative immunoblot analysis of the glial fibrillary acidic protein GFAP in normal versus *mdx*-*4cv* brain. Shown are representative silver-stained gels (**a**, **f**, **i**) and immunoblots (**b**, **c**, **g**, **h**, **j**, **k**). *Panels* **a**–**c** contain 12-month old wild type (*wt*) and *mdx*-*4cv* brain specimens, and *panels* **f**–**k** show 2-, 8- and 12-month old samples. *Blots* were labelled with antibodies to lactate dehydrogenase (*LDH* as loading control) (**b**, **g**, **j**), and the glial fibrillary acidic protein (GFAP) (**c**, **h**, **k**). *Arrowheads* mark the main immuno-labelled protein bands in individual panels. Graphical representations of the immuno-decoration levels for LDH and GFAP in 12-month old *wt* versus *mdx*-*4cv* brain are shown in *panels* **d** and **e**: Student’s *t* test, unpaired; n = 4; *p < 0.05

**Fig. 6:**
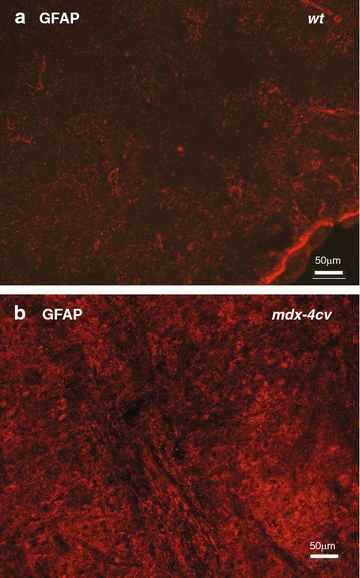
Immunofluorescence microscopical analysis of the glial fibrillary acidic protein in normal versus *mdx*-*4cv* brain. Shown is the immunofluorescence labelling of brain sections using an antibody to glial fibrillary acidic protein (GFAP) in wild type **a** versus *mdx*-*4cv* **b** cortex. The *mdx*-*4cv* cortex exhibited a substantially higher level of GFAP labelling, which agrees with the findings from the mass spectrometric analysis and immunoblotting of the *mdx*-*4cv* brain. *Bar* equals 50 μm

The expression changes of GFAP in *mdx*-*4cv* brain tissue, as indicated by comparative proteomic profiling (Table 1), was confirmed by immunoblotting as illustrated in Fig. 5. The concentration of GFAP was found to be significantly elevated in the *mdx*-*4cv* brain. Standard gel electrophoretic analyses showed no major changes in the protein band pattern of wild type versus dystrophic preparations (Fig. 5a, f, i). In contrast to the expression of lactate dehydrogenase (used as a loading control), which does not appear to be affected in the Dp427-deficient brain (Fig. 5b, d, g, j), the abundance of the glia marker GFAP was shown to be significantly increased in 2- to 12-month old *mdx*-*4cv* mice (Fig. 5c, e, h, k). The cell biological analysis of GFAP levels by immunofluorescence microscopy also confirmed the proteomic analysis of the *mdx*-*4cv* brain. As shown in Fig. 6, the cortex displayed a substantially higher labelling of GFAP in the dystrophin-deficient brain as compared to wild type. These immunoblotting and immunofluorescence microscopical results substantiated the proteomic findings presented in Table 1 and agree with the idea of progressive astrogliosis as a consequence of progressive neurodegeneration in the *mdx*-*4cv* brain. Thus, the GFAP elevation probably reflects non-specific reactive gliosis [69–71] and the resulting glial scar formation may be linked to impaired brain functions in the *mdx* mouse and Duchenne patients [46]. The constant loss of neurons and concomitant proliferation or hypertrophy of glial cells may be the underlying mechanism that triggers developmental cognitive deficits and behavioural abnormalities in Duchenne muscular dystrophy [45–47]. A previous case report of a Duchenne patient with severe mental retardation has described multifocal glial nodules in the cerebral cortex. Proliferating glial cells showed intense immunoreactivity for GFAP and were suggested to be at least partially involved in the pathogenic mechanisms of mental retardation [76].

### Proteomic profiling suggests abnormal calcium handling, cytoskeletal restructuring and metabolic changes in the *mdx*-*4cv* brain

**Fig. 7:**
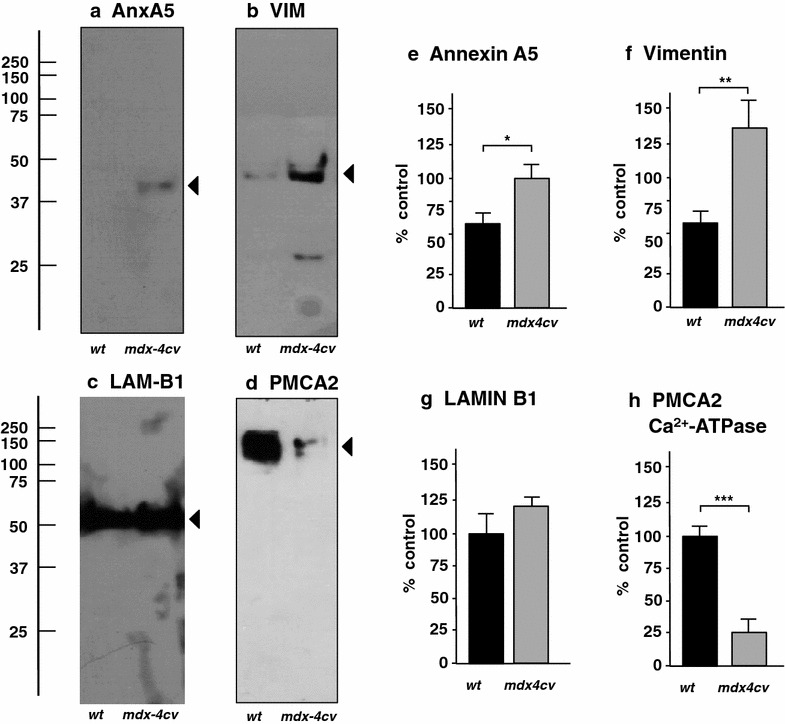
Comparative immunoblot analysis of normal versus *mdx*-*4cv* brain. Shown are representative immunoblots (**a**–**d**) of key proteomic hits identified by mass spectrometric analysis. *Lanes* *1* and *2* represent total extracts from control wild type (*wt*) and *mdx*-*4cv* brain, respectively. *Blots* were labelled with antibodies to the AnxA5 isoform of annexin (**a**), the intermediate filament protein vimentin (VIM) (**b**), the nuclear envelope protein lamin B1 (LAM-B1) (**c**) and the plasma membrane Ca^2+^-ATPase isoform PMCA2 (**d**). *Arrowheads* mark the main immuno-labelled protein bands in individual *panels*. Graphical representations of the immuno-decoration levels are shown in *panels* **e**–**h**: Student’s *t*-test, unpaired; n = 4; *p < 0.05; **p < 0.01; ***p < 0.001

The previous biochemical and cell biological characterization of the brain from dystrophic *mdx* mice has demonstrated intricate changes in the development of the blood–brain barrier [62, 77], the regulation of the extracellular matrix [78] and the molecular arrangements within the glial dystrophin-associated signalling complex that contains the water channel aquaporin AQP4, the K^+^-channel Kir-4.1 and agrin [79]. This suggests multifaceted cellular rearrangements within the dystrophin-lacking brain and the proteomic findings presented here have confirmed this complexity of pathobiochemical alterations. Noteworthy brain tissue-associated proteins with an increased concentration in the *mdx*-*4cv* animal model, besides the glial fibrillary acidic protein GFAP, were identified as the AnxA5 isoform of annexin, the intermediate filament protein vimentin and the nuclear envelope protein lamin LAM-B1. The immunoblot analysis presented in Fig. 7a–c, e–g confirmed the general tendency of higher levels of these proteins, although only changes in annexin and vimentin were shown to be significant. In analogy to findings from dystrophic *mdx* skeletal muscles [80], the up-regulation of annexin probably represents a subcellular repair mechanism involved in membrane maintenance [81] and elevated vimentin levels could function as cytoskeletal stabilization in the absence of brain dystrophin. Alternatively, vimentin changes may be linked to astrogliosis-related alterations in the intermediate filament system. Astrocyte activation and reactive gliosis in the damaged central nervous system has previously shown to be associated with the increased production of cytoskeletal proteins and rearrangements within the intermediate filament system of astrocytes [82].

In addition, the proteomic profiling of the *mdx*-*4cv* brain indicated metabolic adaptations, disturbed ion handling and functional changes in the central nervous system from the dystrophic phenotype. The immunoblotting analysis of the plasma membrane Ca^2+^-transporting ATPase 2 (Fig. 7d, h), which is mainly expressed in brain cortex, confirmed the proteomic identification of significantly reduced PMCA2 levels (Table 1). The Ca^2+^-pump activities of PMCA2 complexes are critical regulators of dendritic ion homeostasis that controls Purkinje cell dendritic growth [83]. In contrast to PMCA2, the Ca^2+^-binding protein calretinin [84] was shown to be significantly increased in the *mdx*-*4cv brain* (Table 1), possibly compensating abnormal calcium fluxes within specific neurons of the central nervous system. Interesting proteomic biomarker candidates with a differential expression pattern are the neuron-specific enzyme ubiquitin carboxyl-terminal hydrolase isozyme L1, the dendritic spine protein drebrin, the cytomatrix protein bassoon of the nerve terminal active zone, and the synapse-associated protein SAP97, as well as the nervous system-specific proteins syntaxin-1B and syntaxin-binding protein 1 [85].

## Conclusions

The comparative proteomic profiling of wild type versus *mdx*-*4cv* brain extracts has resulted in the biochemical identification of a large number of proteins with a changed concentration. These novel proteomic candidates can now be evaluated for their suitability to establish a robust biomarker signature of brain pathology in Duchenne muscular dystrophy. In the future, brain-associated or brain-released protein biomarkers might be useful as predictive, diagnostic, prognostic and/or therapy-monitoring detection tools for the swift evaluation of brain abnormalities in dystrophinopathies. The scale of proteome-wide changes described in this report suggests complex molecular and cellular changes in the *mdx*-*4cv* brain that appear to be linked to both progressive neuron degeneration and reactive gliosis. Of special interest is the proteomic identification of the glial fibrillary acidic protein GFAP, which presents a major intermediate filament protein of mature astrocytes and an established biomarker of astrogliosis. Hence, its significant increase in the *mdx*-*4cv* brain agrees with the histopathological concept of reactive gliosis in the Dp427-deficient central nervous system. In addition, the up-regulation of the annexin isoform AnxA5 and vimentin suggest compensatory mechanisms at the level of plasma membrane repair mechanisms and cytoskeletal re-stabilization. Disturbed neuronal proteins involved in Ca^2+^-handling, metabolism and signalling in the central nervous system illustrate the complexity of the molecular pathogenesis in the dystrophic phenotype.

## Methods

### Materials

Whole tissue proteomics for the comparative profiling of *mdx*-*4cv* versus wild type brain was conducted using analytical grade reagents and materials obtained from GE Healthcare (Little Chalfont, Buckinghamshire, UK) and Bio-Rad Laboratories (Hemel-Hempstead, Hertfordshire, UK). A number of other chemicals were used in this mass spectrometry-based survey, including ultrapure acrylamide stock solutions which were purchased from National Diagnostics (Atlanta, GA, USA), sequencing grade modified trypsin and Lys-C obtained from Promega (Madison, WI, USA) and Whatman nitrocellulose transfer membranes from Invitrogen (Carlsbad, CA, USA). The chemiluminescence substrate and protease inhibitors were purchased from Roche Diagnostics (Mannheim, Germany). Superfrost Plus positively-charged microscope slides were from Menzel Glaesser (Braunschweig, Germany). Primary antibodies were purchased from Abcam, Cambridge, UK (ab14196 to annexin Anx5; ab7260 to glial fibrillary acidic protein GFAP; ab16048 to lamin-B1; ab3529 to the Ca^2+^-ATPase PMCA2; and ab52488 to lactate dehydrogenase) and Dako (Agilent Technologies), Hamburg, Germany (Rabbit polyclonal antibodies Z033429 to glial fibrillary acidic protein GFAP and A0082 to von Willebrand factor vWF). Chemicon International (Temecula, CA, USA) provided peroxidase-conjugated secondary antibodies. Normal goat serum and Cy3-conjugated goat anti-rabbit antibodies were from Jackson ImmunoResearch (West Grove, PA, USA). A range of other general chemicals were used, all of which were analytical grade and were obtained from Sigma Chemical Company (Dorset, UK).

### Dystrophic *mdx*-*4cv* mouse model of X-linked dystrophinopathy

A deeper understanding of the pathobiochemistry of Duchenne muscular dystrophy has been greatly enhanced by the systematic proteomic profiling of established animal models [54]. The conventionally used *mdx* mouse [86] in particular has been extensively used [87]. This genetic model, in analogy to the human condition, is almost completely lacking the full-length Dp427 isoform of dystrophin. Behavioural studies have shown significant differences in the retention of the passive avoidance response [48], long delays in spontaneous alternation and bar-pressing tasks [49] and impaired long-term spatial and recognition memory [50] in the *mdx* mouse. The impairment in passive avoidance learning and behavioural changes in *mdx* mice indicates that the loss of brain dystrophin is associated with cognitive dysfunctions. In relation to behavioural/neurological aspects of the *mdx*-*4cv* strain, the Mouse Genome Information site from Jackson Laboratory states that this dystrophic mouse model exhibits abnormal grip strength and that the determined grip strength is weaker than that of wild type and the *mdx*-*3cv* mouse (http://www.informatics.jax.org). This analysis indicates that muscular weakness and potentially behavioural deficits exist in the *mdx*-*4cv* mouse model of Duchenne muscular dystrophy. Importantly, the *mdx*-*4cv* mouse [88] displays ten-fold fewer revertant dystrophin-positive fibres [89] and immunoblotting has shown the absence of the shorter Dp140 and Dp260 isoforms, as well as the full-length brain, Purkinje and muscle Dp427 isoforms [90]. This renders the *mdx*-*4cv* mouse a more suitable model system for the study of new therapeutic avenues, such as exon skipping or stop-codon read-through therapy [91]. In order to investigate the effect of Dp427 deficiency on protein expression patterns in the central nervous system, whole brains were removed from 2-, 8- and 12-month old *mdx*-*4cv* mice and aged-matched control C57BL6 mice. All animals used in this study were housed at the Bioresource Unit at the University of Bonn [92], where they were kept under standard conditions according to German and Irish legislation on the use of animals in experimental research. The animals were sacrificed by cervical dislocation and all tissue samples were immediately isolated [93].

### Preparation of tissue extracts from *mdx*-*4cv* and wild type brain for proteomic analysis

Brain samples from 12-month old wild type (*wt*) (n = 4) and *mdx*-*4cv* (n = 4) animals were freshly dissected, quick-frozen in liquid-nitrogen and stored at −80 °C prior to usage. Freshly thawed brain tissue (100 mg) was finely chopped and homogenised in 10 volumes of homogenisation buffer (8 M urea, 50 mM Tris–HCl pH 8.0, 1 mM EDTA) using a hand-held IKA T10 Basic Homogeniser (IKA Labortechnik, Staufen, Germany). To limit potential protein degradation by protease activity, this buffer was supplemented with a protease inhibitor cocktail from Roche Diagnostics (Mannheim, Germany). Brain homogenates were incubated at 4 °C for 1.5 h with gentle shaking using a Thermomixer from Eppendorf (Hamburg, Germany). Samples were centrifuged at 14,000×*g* for 20 min at 4 °C, and the urea-soluble protein-containing middle layer was isolated for proteomic analysis [94].

### Preparation of tissue extracts from liver, diaphragm, leg muscle, heart and brain

In order to investigate the tissue-specificity of the antibody to GFAP, comparative immunoblotting was employed. For this purpose a variety of mouse tissue types were used, including liver, heart, hind limb skeletal muscles, diaphragm and brain. All samples were from control wild type mice. For the preparation of total extracts from liver, skeletal muscle and diaphragm, 50 mg of tissue samples were finely chopped and homogenised in 0.5 ml of homogenisation buffer (20 mM sodium pyrophosphate, 20 mM sodium phosphate, 1 mM MgCl_2_, 0.303 M sucrose, 0.5 mM EDTA, pH 7.0), using a hand-held IKA T10 Basic Homogeniser (IKA Labortechnik, Staufen, Germany) [93]. Similarly, 50 mg of heart and brain samples were finely chopped and homogenised in lysis buffer (7 M urea, 2 M thiourea, 4 % CHAPS, 2 % DTT, 2 % IPG buffer pH 3–10) and homogenisation buffer (8 M urea, 50 mM Tris–HCl pH 8.0, 1 mM EDTA), respectively [94]. All buffers were supplemented with a protease inhibitor cocktail from Roche Diagnostics (Mannheim, Germany) to minimise protein degradation. Protein extracts were gently shaken at 4 °C for 1.5 h using a Thermomixer from Eppendorf (Hamburg, Germany). Following centrifugation at 14,000*g* for 20 min at 4 °C, the supernatant fractions were isolated and the protein concentrations were determined by the method of Bradford [95]. Samples were then used for comparative immunoblotting.

### Sample preparation for label-free liquid chromatography mass spectrometry

Prior to mass spectrometric analysis, protein samples were pre-treated with the Ready Prep 2D clean up kit from Bio-Rad Laboratories (Hemel-Hempstead, Hertfordshire, UK). This removed contaminating agents, which may otherwise interfere with the mass spectrometric analysis. The pellets obtained from this procedure were resuspended in label-free solubilisation buffer (6 M urea, 2 M thiourea, 10 mM Tris, pH 8.0 in LC–MS grade water), and samples were vortexed and sonicated to aid full resuspension [93]. Protein concentrations were determined by the method of Bradford [95] and sample volumes were equalised with label-free solubilisation buffer. Samples were treated with 10 mM DTT for 30 min at 37 °C as a means of reducing protein disulphide bonds. Cysteine residues were alkylated by adding iodoacetamide to a concentration of 25 mM in 50 mM ammonium bicarbonate and incubating for 20 min in the dark. To limit the possibility of trypsin alkylation by unreacted iodoacetamide, samples were further reduced with 10 mM dithiothreitol for 15 min in the dark. Protein digestion was carried out in two steps. Firstly proteolytic digestion was carried out with sequencing grade Lys-C at a ratio of 1:100 (protease:protein) at 37 °C for 4 h with agitation. Following the initial cleavage of peptide bonds, samples were diluted with four times the initial sample volume in 50 mM ammonium bicarbonate. The final stage of protein digestion was achieved by incubation with sequencing grade trypsin overnight at 37 °C at a ratio of 1:25 (protease:protein). Acidification with 2 % trifluoroacetic acid (TFA) in 20 % acetonitrile (ACN) [3:1 (v/v) dilution] terminated protein digestion. The sample digests were purified with Pierce C18 Spin Columns from Thermo Fisher Scientific (Dublin, Ireland), dried through vacuum centrifugation and resuspended in loading buffer consisting of 2 % ACN and 0.05 % TFA in LC–MS grade water. To ensure the even suspension of peptides, samples were vortexed and sonicated prior to loading on the mass spectrometer.

### Label-free liquid-chromatography mass spectrometric analysis

The nano LC–MS/MS analysis of *mdx*-*4cv* versus *wt* brain tissue was carried out with an Ultimate 3000 nanoLC system (Dionex) coupled to an LTQ Orbitrap XL mass spectrometer (Thermo Fisher Scientific) in the Proteomics Facility of the National Institute for Cellular Biotechnology, Dublin City University, using optimised methodology [96]. Digested peptide samples (5 µl volume) were loaded onto a C18 trap column (C18 PepMap, 300 μm id × 5 mm, 5 μm particle size, 100 Å pore size; Dionex). Desalting was performed at a flow rate of 25 μl/min in 0.1 % TFA/2 % ACN for 10 min. The trap column was then switched on-line with an analytical PepMap C18 column (75 μm id × 500 mm, 3 μm particle and 100 Å pore size; Dionex). Brain-tissue derived peptides were eluted with the following binary gradients: solvent A [2 % (v/v) ACN (acetonitrile) and 0.1 % (v/v) formic acid in LC–MS grade water] and 0–25 % solvent B [80 % ACN and 0.08 % (v/v) formic acid in LC–MS grade water] for 240 min and 25–50 % solvent B for a further 60 min, with a column flow rate set to 350 nl/min [86]. Data were acquired with Xcalibur software, version 2.0.7 (Thermo Fisher Scientific). The MS apparatus was operated in positive, data-dependent mode and was externally calibrated. Survey MS scans were attained in the 400–1200 *m*/*z* range with the resolution set to a value of 30,000 at *m*/*z* 400 and lock mass set to 445.120025u. Collision-induced dissociation (CID) fragmentation was carried out with the three most intense ions per scan. A dynamic exclusion window was applied within 60 s [97]. A normalized collision energy of 35 %, an isolation window of 3 *m*/*z* and one microscan were used to collect suitable tandem mass spectra.

### Quantitative mass spectrometric identification of brain-derived proteins

The raw data obtained from the LC–MS/MS analysis was processed using Progenesis QI for Proteomics software (version 2.0; Non-Linear Dynamics, a Waters company, Newcastle upon Tyne, UK). Crucially the LC retention times of all data were aligned to an assigned reference run (run with the most peptides), to allow for any drift in retention time [95]. Prior to exportation to Proteome Discoverer 1.4 (Thermo Scientific), the MS/MS data files were filtered using the following parameters; (1) peptide features with ANOVA ≤ 0.05 between experimental groups, (2) mass peaks with charge states from +1 to +5 and (3) greater than one isotope per peptide. The PepXML generic file, generated from all exported MS/MS spectra, was used for peptide identification using Proteome Discoverer 1.4 against Mascot (version 2.3, Matrix Science, Boston, MA, USA) and Sequest HT (SEQUEST HT algorithm, licence Thermo Scientific, registered trademark University of Washington, USA) and searched against the UniProtKB-SwissProt database (taxonomy: *Mus musculus*). The following search parameters were used for protein identification: (1) peptide mass tolerance set to 20 ppm, (2) MS/MS mass tolerance set to 0.6 Da, (3) up to two missed cleavages were allowed, (4) carbamidomethylation set as a fixed modification and (5) methionine oxidation set as a variable modification [97]. For re-importation back into Progenesis LC–MS software for further analysis, only peptides with either ion scores of 40.00 or more (from Mascot) and peptides with XCorr scores >1.9 for singly charged ions, >2.2 for doubly charged ions and >3.75 for triply charged ions or more (from Sequest HT) were selected. A number of criteria were applied to ensure proper identification of brain-tissue derived proteins, including an ANOVA score between experimental groups of ≤0.05 and proteins with ≥2 peptides matched.

### Bioinformatics analysis of potential protein interactions

The freely available software package PANTHER (http://pantherdb.org) was used for the bioinformatics analysis of the mass spectrometric findings generated in this study [68]. Proteins with an altered expression in *mdx*-*4cv* brain samples were grouped based on their protein class using the PANTHER database of protein families.

### Independent verification of key proteomic hits by immunoblot analysis

In order to confirm alterations in protein expression, as identified by label-free mass spectrometry, immunoblot analysis using a panel of antibodies against select proteins was employed. Electrophoretic separation of proteins from 2-, 8- and 12-month old wild type and *mdx*-*4cv* brain tissues, as well as extracts from liver, heart, diaphragm and leg muscles, was performed using standard 10 % polyacrylamide gels, followed by wet transfer at 100 V for 70 min at 4 °C to Whatman Protan nitrocellulose sheets in a Transblot Cell from Bio-Rad Laboratories (Hemel-Hempstead, Hertfordshire, UK) as per standard procedure [98]. Membranes were blocked for 1 h at room temperature with a milk protein solution [2.5 % (w/v) fat-free milk powder in 10 % phosphate-buffered saline] prior to incubation with primary antibodies to limit non-specific binding. Nitrocellulose sheets were then incubated with sufficiently diluted primary antibodies overnight at 4 °C with gentle agitation. Membranes were subsequently washed twice with the milk protein solution for 10 min each time, followed by incubation for 1.5 h with peroxidase-conjugated secondary antibodies, diluted in the blocking buffer. Visualisation of antibody-labelled protein bands on washed membranes was achieved using enhanced chemiluminescence as per manufacturer’s guidelines. Densitometric scanning and statistical analysis of immunoblots was performed with a HP PSC-2355 scanner and ImageJ software (NIH, USA), in conjunction with GraphPad Prism software (San Diego, CA, USA), in which a p value <0.05 was deemed to be statistically significant.

### Immunofluorescence microscopy of brain sections

In order to verify the increased abundance of the glial fibrillary acidic protein (GFAP) in the Dp427-deficient brain, as well as study vascularization by labelling brain tissue with von Willebrand factor (vWF), *mdx*-*4cv* versus wild type cortex sections were analysed by immunofluorescence microscopy [32]. Brains were carefully removed and quick-frozen in liquid nitrogen. Cryo-sectioning was used to produce 10 µm sagittal sections, which were placed on Superfrost Plus positively-charged microscope slides. Brain sections were fixed for 5 min in ice-cold 4 % (v/v) paraformaldehyde in phosphate-buffered saline (PBS). Sections were then permeabilized in 0.1 % (v/v) Triton X-100 in PBS for 30 min at room temperature. Tissue sections were blocked in 20 % (v/v) normal goat serum in PBS for 30 min and incubated overnight at 4 °C with rabbit polyclonal antibodies to GFAP (1:300 in PBS) or vWF (1:400 in PBS). Following a careful washing step, brain sections were incubated with Cy3-conjugated anti-rabbit antibodies (1:300) for 30 min at room temperature. Primary antibodies were omitted for control staining. Antibody-labelled brain sections were embedded in Fluoromount G medium and viewed under a Zeiss Axioskop 2 epi-fluorescence microscope equipped with a digital Zeiss AxioCam HRc camera (Carl Zeiss Jena GmbH, Jena, Germany).
